# Protective Effects of Let-7a and Let-7b on Oxidized Low-Density Lipoprotein Induced Endothelial Cell Injuries

**DOI:** 10.1371/journal.pone.0106540

**Published:** 2014-09-23

**Authors:** Mei-hua Bao, Yi-wen Zhang, Xiao-ya Lou, Yu Cheng, Hong-hao Zhou

**Affiliations:** 1 Department of Human Anatomy, Histology and Embryology, Institute of Neuroscience, Changsha Medical University, Changsha, China; 2 Department of Clinical Pharmacology, Xiangya Hospital, Central South University, Changsha, P. R. China; Institute of Clinical Pharmacology, Central South University; Hunan Key Laboratory of Pharmacogenetics, Changsha, P. R. China; Albany Medical College, United States of America

## Abstract

Lectin-like low-density lipoprotein receptor 1 (LOX-1) is a receptor for oxidized low density lipoprotein (oxLDL) in endothelial cells. The activation of LOX-1 by oxLDL stimulates the apoptosis and dysfunction of endothelial cells, and contributes to atherogenesis. However, the regulatory factors for LOX-1 are still unclear. MicroRNAs are small, endogenous, non-coding RNAs that regulate gene expressions at a post-transcriptional level. The let-7 family is the second microRNA been discovered, which plays important roles in cardiovascular diseases. Let-7a and let-7b were predicted to target LOX-1 3′-UTR and be highly expressed in endothelial cells. The present study demonstrated that LOX-1 was a target of let-7a and let-7b. They inhibited the expression of LOX-1 by targeting the positions of 310-316 in LOX-1 3′-UTR. Over-expression of let-7a and let-7b inhibited the oxLDL-induced endothelial cell apoptosis, NO deficiency, ROS over-production, LOX-1 upregulation and endothelial nitric oxide synthase (eNOS) downregulation. Moreover, we found that oxLDL treatment induced p38MAPK phosphorylation, NF-κB nuclear translocation, IκB degradation and PKB dephosphorylation. Let-7a or let-7b over-expression attenuated these alterations significantly. The present study may provide a new insight into the protective properties of let-7a and let-7b in preventing the endothelial dysfunction associated with cardiovascular disease, such as atherosclerosis.

## Introduction

OxLDL-induced endothelial cell apoptosis and dysfunction play key roles in the pathogenesis of atherosclerosis [1], [2], . Lectin-like low-density lipoprotein receptor 1 (LOX-1) is a multifunctional receptor participating in cardiovascular dysfunction [4]. It was first identified to be a receptor for oxLDL in endothelial cells [5]. The activation of LOX-1 by oxLDL stimulates the inflammation, apoptosis and dysfunction of endothelial cells, and promotes the pathogenesis of atherosclerosis [2], [6], [7], [8], [9]. Previous studies have found that the suppression of LOX-1 is beneficial to the cardiovascular system [10], [11], [12].

MicroRNAs are small, endogenous, non-coding RNAs that regulate gene expressions in post-transcriptional level. The let-7 family, consisting of 9 members, is the second discovered microRNA, which was found in *C. elegans* [13]. Recent research has found that it is highly expressed in the cardiovascular system, and plays an important role in cardiovascular diseases as well as in heart development [14], [15], [16]. Let-7's levels were found to be related to atherosclerosis and coronary artery disease. Let-7g was demonstrated to target LOX-1 3′-UTR and participate in oxLDL-induced vascular smooth muscle cell (VSMC) proliferation and migration [17], autophagy, and apoptosis [15]. However, besides let-7g, let-7a and let-7b were also predicted to target LOX-1 3′-UTR by bioinformatics tools. Moreover, they share the same binding site in LOX-1 3′-UTR, and are highly expressed in endothelial cells [18]. Therefore, we hypothesize that let-7a and let-7b may target LOX-1 3′-UTR and regulate the expression of LOX-1. They may also play an important role in oxLDL-induced endothelial cell injuries. To validate this hypothesis, the present study was performed to investigate whether or not LOX-1 is one of the targets for let-7a and let-7b, and to examine the anti-apoptotic effects and some of the mechanisms of let-7a and let-7b on oxLDL treated endothelial cells.

## Materials and Methods

### Materials

HUVEC cell line and HEK293 cells were purchased from the American Type Culture Collection (Manassas VA, USA); oxidized low-density lipoprotein was supplied by Beijing Xiesheng Biological Technology Co., Ltd (Beijing, China); SYBR Premix DimerEraser (Perfect Real Time) assay kit and PrimeScript RT reagent Kit With gDNA Eraser (Perfect Real Time) kit were purchased from Takara (Dalian, China); the primers were provided by Shanghai Sangon Biological Engineering Co. Ltd; let-7a and let-7b mimics, inhibitors and primers were obtained from RiboBio Co., Ltd (Guangzhou, China), Dual-luciferase reporter assay system kit was provided by Promega (Madison, USA); LDH assay kit, NO assay kit and DCFH-DA were obtained from Beyotime (Shanghai, China). Secrete-Pair Dual Luminescence Assay Kit was provided by GeneCopoeia Inc.(Rockville, USA); rabbit polyclonal antibody to LOX-1 was provided by Abcam (New Territories, HK, ab60178); mouse anti-p38MAPK monoclonal antibody (sc-81621), mouse anti-p-p38MAPK monoclonal antibody (sc-7973), mouse anti-NF-κB(p65) polyclonal antibody (sc-372), mouse anti-IκBα monoclonal antibody (sc-373893), rabbit polyclonal antibody to eNOS (SC-654), rabbit polyclonal antibody to Histon H3 (sc-8654), second antibody were provided by Santa Cruz Biotechnology Inc.(Texas, USA). Rabbit anti-PKB monoclonal antibody (C67E7), and rabbit anti-p-PKB monoclonal antibody (S473) were obtained from Cell Signaling Technology Inc. (Massachusetts, USA).

### Cell culture and transfection

HUVECs were grown in DMEM (low glucose) supplemented with 10% fetal bovine serum, and maintained in a humidified atmosphere containing 5% CO_2_ at 37°C. The cells between passages 2 and 15 were used in this study.

For the transfection, different amount of lipofectamin 2000, microRNA mimics, microRNA inhibitors (or NC mimics, NC inhibitor), or LOX-1 3′-UTR reporter plasmids were diluted in Opti-MEM I Medium (invitrogen) and incubated for 5 min. After incubation, transfection complexes were obtained by mixing equal parts of the diluted lipofectamin 2000 and the diluted microRNA mimics (or microRNA inhibitors), and incubated for another 20 min. These transfection complexes were then added to cells and incubated for 6 hours, media was then replaced by fresh normal growth medium, and incubated for another 24 hours.

### Computational analysis of let-7a and let-7b binding sites in LOX-1 3′-UTR

The binding sites for let-7a and let-7b in 3′-UTR region of LOX-1 were analyzed by pictar: http://pictar.mdc-berlin.de/, targetscan: http://www.targetscan.org/, and miRBASE: http://www.mirbase.org/.

### LOX-1 3′-UTR (wild type and mutant) reporter plasmids construction, transfection and dual luciferase reporter analysis

PCR was performed using primers specific for the LOX-1 3′-UTR (forward primer 5′-CCG CTC GAG AGT CCG AGG CGC TGT CTC C -3′, reverse primer 5′- GAA TGC GGC CGC TCT TGT ACA CAA ATG TTC ACA GCA GC -3′). The forward primers included an Xhol cutting site, and the reverse primer included a Notl cutting site. 293T cell's genomic DNA was used as the template. PCR products were then digested with Xhol and Notl and cloned to pmir-REPORT luciferase vector (Ambion). The mutation at the potential binding site of let-7 was performed using the QuikChange Site-Directed Mutagenesis Kit (Stratagene, USA). The predicted let-7 binding sites (underline) were changed as follows: wild type, 5′-TTC TTT ACC TCA TTA TCA CCT TCC CCT CAC AC-3′; mutant, 5′- TTC TTT **CAT GAA C**TA TCA CCT TCC CCT CAC AC -3′.

For the transfection, HUVECs were planted into a 24-well plate at the density of 5×10^4^ cells/well for 24 hours. 400 ng of wild type, mutant or blank plasmid DNA were co-transfected with let-7a mimics (75 nM), let-7b mimics (75 nM), let-7a/7b mimics (75 nM respectively), NC mimics (75 nM), let-7a/7b inhibitor (100 nM respectively), or NC inhibitor (100 nM). Dual luciferase reporter analysis was performed at 48 hours post-transfection using the Luciferase Assay kit according to the manufacturer's instructions (Promega).

### QPCR and Western-blot for LOX-1 mRNA and protein expression

HUVECs were planted into a 6 well plate at the density of 2×10^5^ cells/well for 24 hours, and transfected with let-7a mimics (75 nM), let-7b mimics (75 nM), let-7a/7b mimics (75 nM respectively), NC mimics (75 nM), let-7a/7b inhibitor (100 nM respectively), or NC inhibitor (100 nM) as described in section **2.2**. After transfection, total RNA were extracted by Trizol, after determination of RNA concentration, the SYBR Premix DimerEraser (Perfect Real Time) assay kits and PrimeScript RT reagent Kit With gDNA Eraser (Perfect Real Time) kit (Takara, China) were used for RT-PCR reaction. The primers for LOX-1: Forward: 5′-GAG AGT AGC AAA TTG TTC AGC TCC TT-3′, Reverse: 5′-GCC CGA GGA AAA TAG GTA ACA GT -3′; β-actin: Forward: 5′-TGA CTG ACT ACC TCA TGA AGA T-3′, Reverse: 5′-CAT GAT GGA GTT GAA GGT AGT T-3′; The protocol for real-time PCR was an initial incubation at 95°C for 30 s, followed by 40 cycles of 95°C 5 s, 60°C 30 s. All samples were run in triplicate. The β-actin was used as an internal reference, and the results were analyzed using the 2^(−ΔΔCt)^ Method.

The LOX-1 proteins were detected by Western-blot as described before [19]. Briefly, the total proteins were separated by 10% SDS-PAGE and transferred to nitrocellulose membranes. After incubation in blocking solution (4% non-fat milk), the membranes were incubated with 1∶500 dilution primary antibodies that detect LOX-1 overnight at 4°C. Membranes were washed and incubated with 1∶10000 dilution of second antibody for 1 h, and were detected with Odyssey Infrared Imaging System. Relative intensities were analyzed by Quantity One v4.62 software.

### Expression alteration of let-7a and let-7b in oxLDL treated endothelial cells

HUVECs were planted into a 6 well plate at the density of 2×10^5^ cells/well for 24 hours, and treated with different concentration (50, 100, 150 µg/ml) of oxLDL for 24 hours, or treated with 150 µg/ml oxLDL for different time (6, 12, 24 hours).

Total RNA were extracted by Trizol. After the determination of the RNA concentration, PrimeScript RT reagent Kit With gDNA Eraser (Perfect Real Time) kit was used for reverse transcription. The RT primers for microRNAs are special stem loop primers, which were purchased from RIBOBIO Inc. For the PCR amplification, the SYBR Premix DimerEraser (Perfect Real Time) assay kits were used. The primers for U6 and let-7a, let-7b were provided by RIBOBIO Inc. The real-time PCR was performed using the Roche LC480 PCR System. The optimization of the amplification reaction was assured by a dissociation curve analysis. The basic protocol for real-time PCR was an initial incubation at 95°C for 30 s, followed by 40 cycles of 95°C 5 s, 60°C 30 s. All samples were run in triplicate, and the U6 was used as internal reference, and the results were analyzed using the 2^(−ΔΔCt)^ Method.

### Cell viability, NO, and LDH content determination

HUVECs were planted into a 24 well plate at the density of 5×10^4^ cells/well for 24 hours, and transfected with 75 nM let-7 mimics or mimics NC as described in **section 2.2**. After transfection, cells were treated with oxLDL (150 µg/ml) for 24 hours. The cell viability was assessed by MTS reagent was added to each well according to the manufacturer's instructions. The cell viability was determined by measuring the absorbance at 490 nm using a plate reader. The LDH released into the media was analyzed using analysis kits according to the manufacturer's instructions. The total NO released to media was determined by total nitric assay kit. Briefly, the nitrate was first reduced to nitrite by nitrate reductase, the total nitrite was then measured by Griess reagent. The absorbance at 540 nm was measured, and NO concentration was determined using a curve calibrated on sodium nitrite standards.

### Hoechst 33342 staining

Hoechst 33342 staining was used to detect the nuclear chromatin morphological changes of apoptotic cells. The cells were seeded in 96-well plates at a density of 1×10^4^ per well, and transfected with 75 nM let-7 mimics or mimics NC as described in section **2.2**. After transfection, cells were treated with oxLDL (150 µg/ml) for 24 hours. After washing the plates twice with PBS, cell fixation was carried out using 4% paraformaldehyde for 15 min. Another two washes with PBS followed the fixation, and the cells were incubated in 50 µl of Hoechst 33342 solution (5 µg/ml in PBS) for 20 min in the dark. Fluorescence microscopy was applied to examine the nuclear DNA staining.

### Flow cytometry

By following the manufacturer's instructions, the Annexin V-FITC kit for flow cytometry was used to measure the percentage of apoptotic cells. Briefly, HUVECs were cultured in 6-well plates. The density of the culturing was 2×10^5^ cells per well. Cells were transfected with 75 nM let-7 mimics or mimics NC as described in section **2.2**, and were afterwards treated with oxLDL (150 µg/ml) for 24 hours. Using trypsinization, cells were harvested, followed by the incubation with annexinV-FITC and propidium iodide (PI) for 15 min at room temperature in the dark. Flow cytometry was then employed to analyze the cell apoptotic rates.

### Intracellular ROS production

HUVECs were planted into a 6-well plate at the density of 2×10^5^ cells/well for 24 hours, and transfected with 75 nM let-7 mimics or mimics NC as described at section **2.2**. After transfection, cells were treated with oxLDL (150 µg/ml) for 4 hours. 2′,7′-dichlorofluorescein diacetate (DCFH-DA) was used to detect the intracellular ROS. After treatment, the cells were incubated for 30 min with DCFH-DA (10 µM) at 37°C. Flow cytometry was used to detect the fluorescence intensity.

### QPCR for LOX-1 mRNA and eNOS mRNA expression

HUVECs were planted into a 6 well plate at the density of 2×10^5^ cells/well for 24 hours, and transfected with 75 nM let-7 mimics or mimics NC as described at section **2.2**. After transfection, cells were treated with oxLDL (150 µg/ml) for 24 hours. The qPCR were performed as described in section **2.5**. The primers used for eNOS: Forward: 5′- TCT CCG CCT CGC TCA T-3′, Reverse: 5′-AGC CAT ACA GGA TTG TCG CC-3′.

### Western-blot analysis for p38MAPK, p-p38MAPK, PKB, p-PKB, IκBα protein expression and NF-κB nuclear translocation

HUVECs were transfected with 75 nM let-7 mimics or mimics NC as described in section **2.2** and then treated with oxLDL (150 µg/ml) for 4 hours. The total cytosol/nulei protein was extracted as described above [19]. 10% SDS-PAGE was used to separate the proteins, which were then transferred to nitrocellulose membranes. The membranes were incubated with various dilutions of antibodies following the incubation in blocking solution (4% non-fat milk), namely, the 1∶200 dilution primary antibodies to detect p38MAPK, p-p38MAPK, PKB, p-PKB, NF-κB, IκBα and 1∶1000 dilution of Histon H3 and β-actin antibody for overnight at 4°C. Incubation of washed membranes with 1∶10000 dilution of second antibody for 1 h was performed, and the membranes were detected with Odyssey Infrared Imaging System. Quantity One v4.62 software was used to analyze relative intensities.

### Western-blot detection for LOX-1 and eNOS protein expression

HUVECs were transfected with 75 nM let-7 mimics or mimics NC as described in section **2.2** and then treated with oxLDL (150 µg/ml) for 24 hours. 10% SDS-PAGE was used to separate the total proteins were separated, which were then transferred to nitrocellulose membranes. Following the incubation in blocking solution (4% non-fat milk), incubation of the membranes with 1∶500 dilution primary antibodies to detect LOX-1 and eNOS was done for overnight at 4°C. Membranes were washed and incubated with 1∶10000 dilution of second antibody for 1 h, and were detected with Odyssey Infrared Imaging System. Relative intensities were analyzed by Quantity One v4.62 software.

### Statistical analysis

Statistics from three independent experiments are given as mean ± S.D. ANOVA followed by Newman-Student-Keuls test was used to determine the significance of the differences. A value of *P*<0.05 is considered statistically significant.

## Results

### Regulatory effects of let-7a and let-7b on LOX-1 3′-UTR plasmids

**Figure 1:**
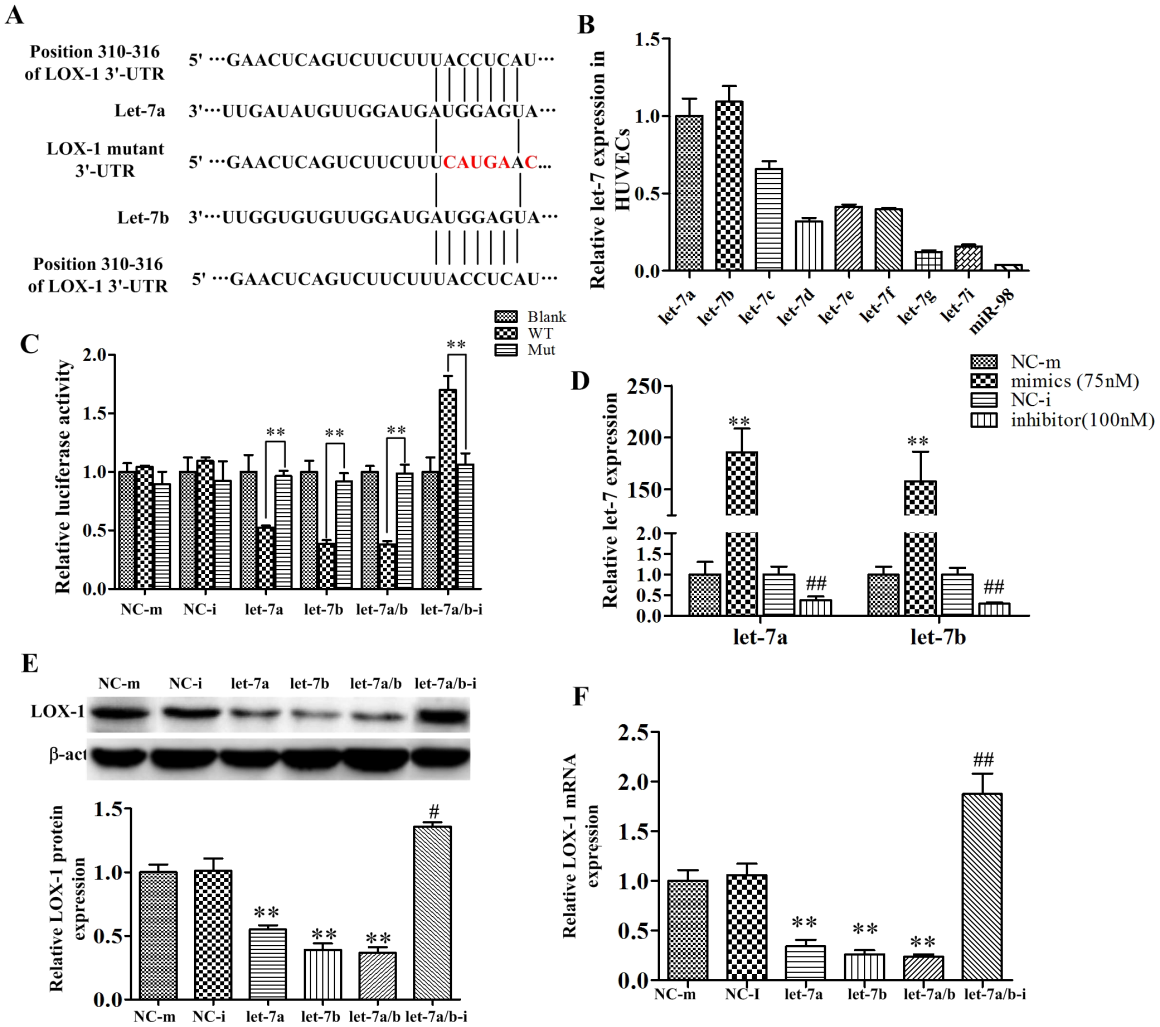
Regulatory effects of let-7 on LOX-1. A: the predicted let-7a and let-7b binding site in LOX-1 3′-UTR (wild type and mutant). B: Relative let-7 family members expressed in HUVECs. C: The relative luciferase activities measured by dual luciferase reporter analysis. HUVECs were planted into 24-well plate at density of 5×10^4^ cells/well for 24 hours. 400 ng of wild type (WT), mutant (MT) or blank plasmid DNA were co-transfected with 75 nM let-7a, let-7b mimics, or 100 nM let-7a/7b inhibitors. Dual luciferase reporter analysis was performed at 48 hours post-transfection using the Luciferase Assay kit according to the manufacturer's instructions. D: The relative let-7a and let-7b expression in HUVECs after transfected with different concentration of mimic negative control (NC-m), let-7a (or let-7b) mimics, inhibitor negative control (NC-i), let-7a (or let-7b) inhibitor or let-7b inhibitor for 48 hours. E: The protein expression of LOX-1. F: The mRNA expression of LOX-1. The HUVECs were transfected with 75 nM let-7a, let-7b mimics or 100 nM let-7a/7b inhibitors for 48 hours and the protein levels were analyzed by Western-blot, the mRNA levels were analyzed by quantative real-time PCR. Data are Mean ± SD from 3 independent experiments, **P<0.01, *P<0.05 compared with NC-m, ^##^ *P*<0.01, ^#^ *P*<0.05 compared with NC-i.

Let-7a and let-7b are predicted to bind to the positions of 310-316 in LOX-1 3′-UTR (Fig. 1A), and they have the same seed sequence. Moreover, in HUVECs, let-7a and let-7b are abundantly expressed (Fig. 1B). We thus tested in the present study whether the positions of 310-316 in LOX-1 3′-UTR form a target gene for let-7. The full length (1597 bp) of LOX-1 3′-UTR was cloned to pmir-REPORT^™^ plasmid, and dual luciferase assay was performed. As shown in Fig. 1C, let-7a, let-7b, let-7a/7b mimics decreased the luciferase activity significantly. Interestingly, when let-7a/7b were co-expressed, the luciferase activity was not lower than that by over-expression of let-7a or let-7b alone, indicating the saturation of let-7 binding sites in LOX-1 gene. On the other hand, the let-7a/7b inhibitor increased the luciferase activity significantly. When the predicted binding position (310-316) of let-7's in LOX-1 3′-UTR was mutant, the effects of let-7a and let-7b were abolished. These results suggested that let-7a and let-7b directly bind to the 310-316 of LOX-1 3′-UTR and inhibit its expression.

### Regulatory effects of let-7a and let-7b on LOX-1 mRNA and protein expression

To validate the effects of let-7a and let-7b on LOX-1 expression, the mimics or the inhibitor of let-7a and let-7b were transiently transfected into HUVECs. After 24 hours of transfection, the let-7a and let-7b expression increased dramatically for 75 nM mimics, and decreased significantly for 100 nM inhibitors (Fig. 1D). The transfection of 75 nM of let-7a and let-7b mimics significantly decreased the LOX-1 mRNA and protein expressions, while the let-7a/7b inhibitor significantly increased LOX-1 mRNA and protein expression (Fig. 1E, F). Thus, the present results validated LOX-1 as a let-7a, and let-7b target gene.

### Effects of oxLDL on let-7a, let-7b and LOX-1 expression in HUVECs

**Figure 2:**
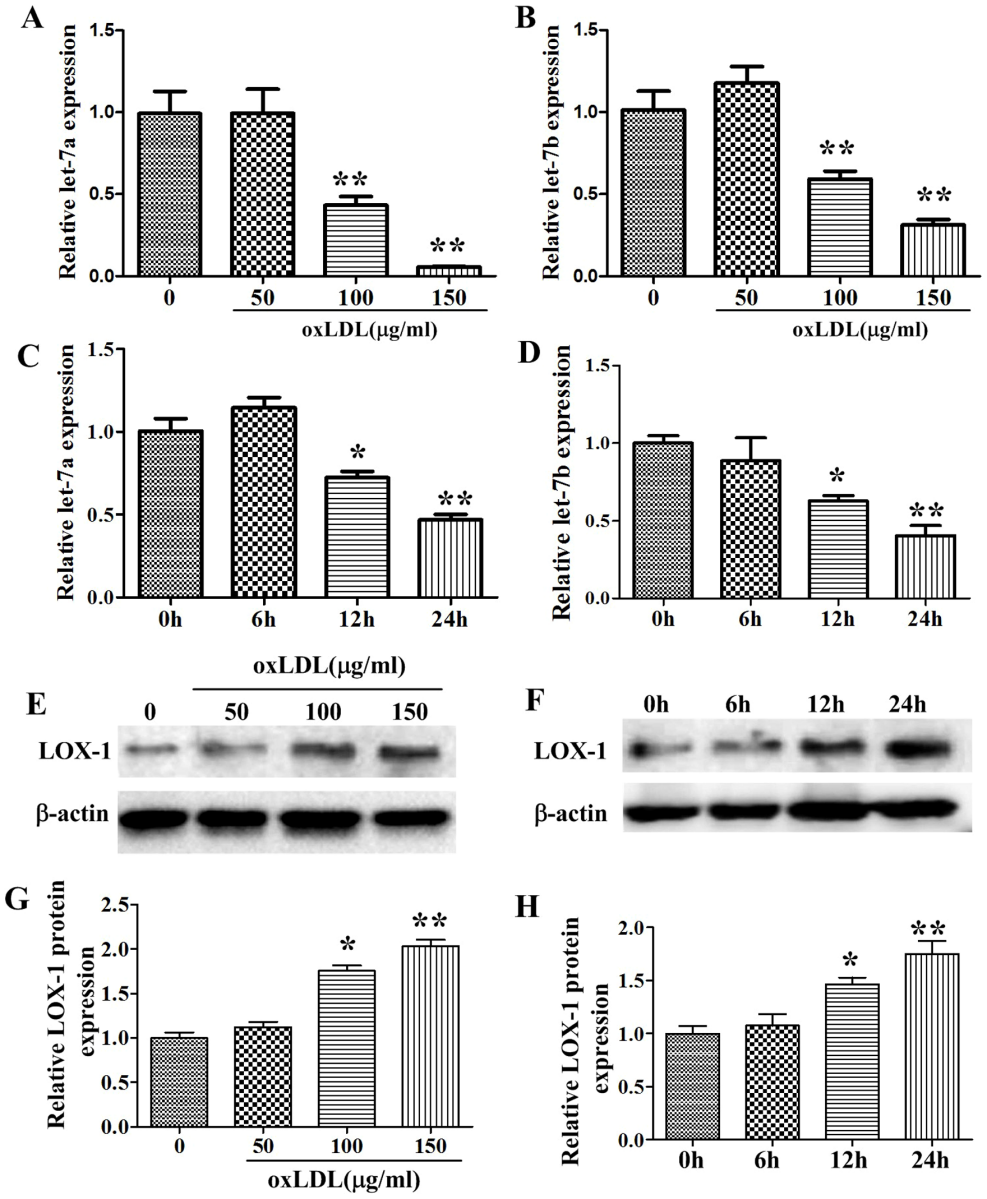
Effects of oxLDL on let-7a, let-7b and LOX-1 expression in HUVECs. A, B: effects of different concentration of oxLDL on let-7a and let-7b expression; C, D: effects of different time treatment with oxLDL (150 µg/ml) on let-7a and let-7b expression; E, G: effects of different concentration of oxLDL on LOX-1 expression; F, H: effects of different time treatment with oxLDL (150 µg/ml) on LOX-1 expression; The values (mean ± SD from three independent experiments) are relative to NC, which was set as 1. *P<0.05, **P<0.01 vs. control.

As shown in Fig. 2A–D, low concentration (50 µg/ml) of oxLDL or short time (6 hours) of oxLDL treatment had no effects on let-7a and let-7b expressions. High concentrations of oxLDL (100, 150 µg/ml) significantly inhibited the expressions of let-7a and let-7b in a concentration-dependent manner. 12 and 24 hours of oxLDL (150 µg/ml) treatment inhibited the level of let-7a and let-7b in a time-dependent manner. These effects of oxLDL were accompanied with the upregulation of LOX-1 expression (Fig. 2E–H).

### Effects of let-7a and let-7b on oxLDL-induced cell viability, LDH, NO alteration

**Figure 3:**
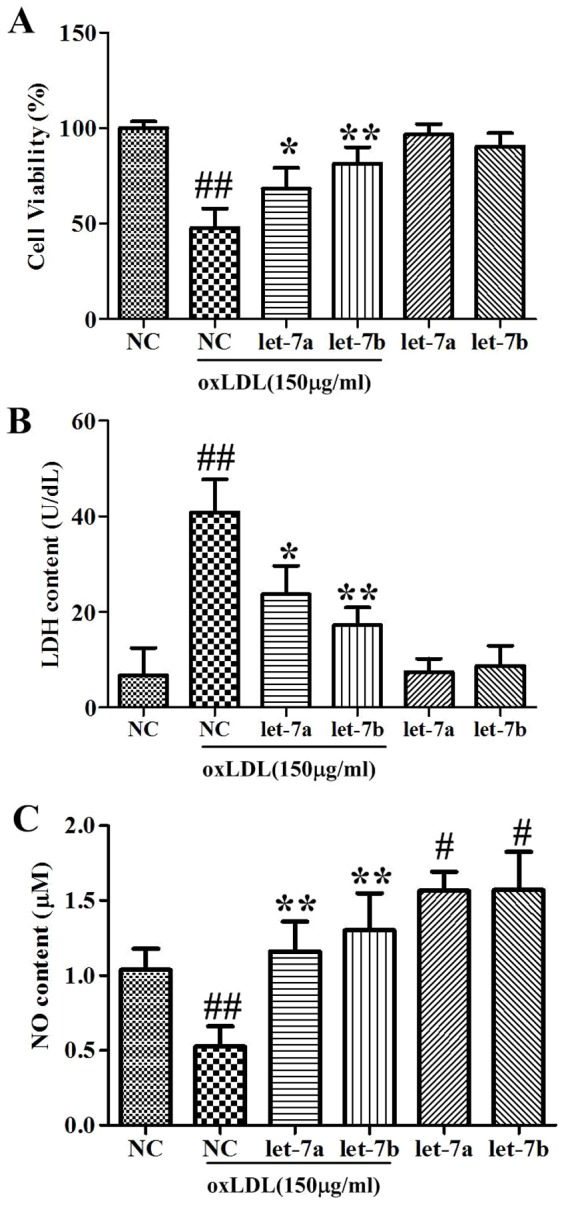
Effects of let-7a and let-7b on oxLDL-induced cell viability, LDH, and NO alteration. A: Cell viability; B: LDH content; C, NO content. The values (mean ± SD from three independent experiments). ^#^ *P*<0.05, ^##^ *P*<0.01 compared with NC, *P<0.05,**P<0.01 compared with oxLDL group.

As shown in Figure 3, oxLDL (150 µg/ml) treatment for 24 hours inhibited the cell viability (47.7% of control), suppressed the NO secretion (52.4% of control), and increased the LDH content (6.06 folds of control). Let-7a and let-7b over-expression inhibited the oxLDL-induced cell injuries. Compared with the oxLDL group, they increased cell viability and NO, and decreased LDH significantly. Interestingly, we found that let-7a or let-7b over-expression alone could increase the NO content significantly. These results indicate that let-7a and let-7b achieve their protective effects through both oxLDL dependent and independent way. Let-7a and let-7b alone did not have effects on cell viability and LDH content.

### Effects of let-7a and let-7b on oxLDL-induced cell apoptosis

**Figure 4:**
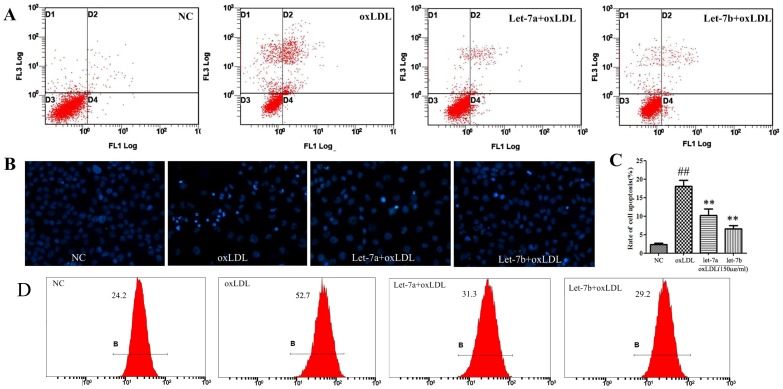
Effects of let-7a and let-7b on oxLDL-induced endothelial cell apoptosis. A: Flow cytometry dot plot figures of apoptotic cells. In each dot plot figure, the upper left quadrant corresponds to necrotic cells; the upper right quadrant contains the later apoptotic cells, which are positive for Annexin V and propidium iodide (PI); the lower left quadrant shows viable cells, which exclude PI and Annexin V; the lower right quadrant represents the early apoptotic cells, Annexin V positive and PI negative. B: Hoechst staining images; C: Rate of apoptotic cells quantified by flow cytometry; D: Effects of let-7a and let-7b on oxLDL-induced ROS production. The values (mean ± SD from three independent experiments). ^##^ *P*<0.01 compared with NC, **P<0.01 compared with oxLDL.

To investigate the effects of let-7a and let-7b on oxLDL induced endothelial cell apoptosis, Hoechst staining and flow cytometry analysis were used in the present study. As shown in Figure 4B, when stained with Hoechest 33342, cells in NC group displayed uniform blue chromatin with organized structure, whereas oxLDL treatment resulted in more frequent apoptotic morphological changes (i.e., a bright blue fluorescent condensed nuclei and chromatin fragmentation, by fluorescence microscope) compared to NC. Treatment of let-7a and let-7b suppressed ox-LDL-induced apoptosis. Further annexin V/PI double staining and flow cytometry analysis confirmed the inhibitory effects of let-7a and let-7b on ox-LDL induced cell apoptosis. The representative images for flow cytometry and the summarized data were presented in Figure 4A. Ox-LDL increased the apoptosis rate from 2.28% to 18.08% compared with control (*P*<0.01). Let-7a and let-7b over-expressions significantly reduced the apoptosis to 10.27% and 6.61% respectively (Fig. 4C).

### Effects of let-7a and let-7b on oxLDL-induced ROS overproduction

The ROS in endothelial cells were detected by DCFH-DA staining and FCM assay. As shown in Figure 4D, the fluorescence intensity in the NC group was 24.2. OxLDL treatment increased the intensity by 2.18 folds, while let-7a and let-7b over-expression inhibited the oxLDL-induced ROS production.

### Effects of let-7a and let-7b on oxLDL-induced LOX-1, eNOS mRNA and protein alteration

**Figure 5:**
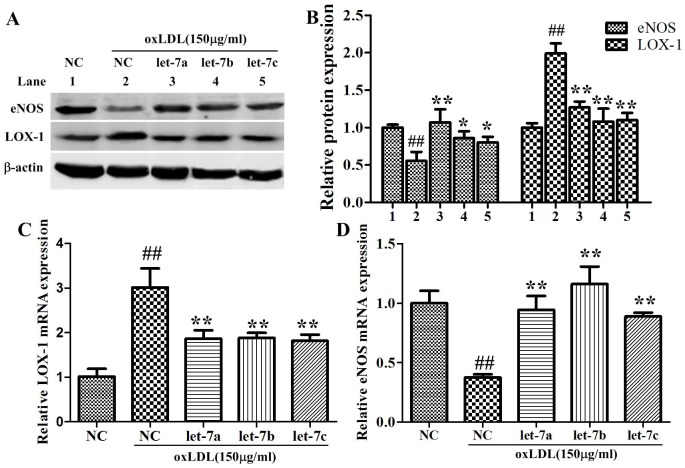
Effects of let-7a and let-7b on oxLDL-induced alteration of LOX-1, eNOS expression. A: LOX-1 and eNOS protein expressions analyzed by Western-blot; C, D: LOX-1 and eNOS mRNA expressions were analyzed by quantitative real-time PCR. The values (mean ± SD from three independent experiments) are relative to negative control, which was set as 1. ^##^ *P*<0.01 vs. NC, *P<0.05; **P<0.01 vs. oxLDL.

As shown in Figure 5, the oxLDL treatment significantly increased the LOX-1 mRNA and protein expression (about 3.01 and 1.89 folds compared to the control, respectively), and suppressed the mRNA and protein expression of eNOS (about 37.4% and 65.6% of the control, respectively). Over-expression of let-7a and let-7b significantly attenuated the oxLDL-induced up-regulation of LOX-1, and the reduction of eNOS expression (*P*<0.05).

### Effects of let-7a and let-7b on p38MAPK and PKB phosphorylation

**Figure 6:**
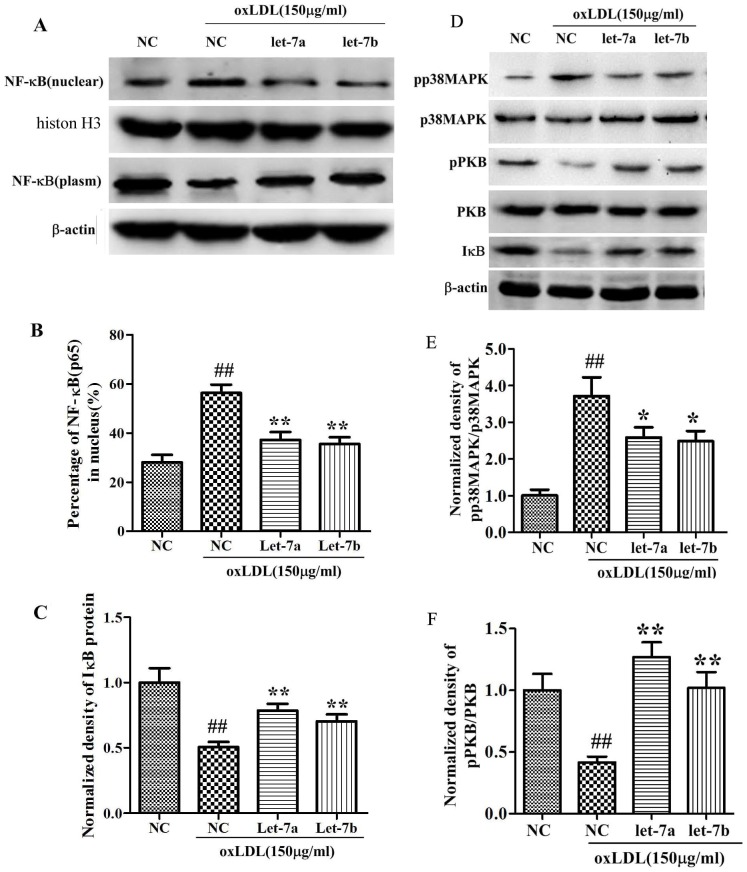
Effects of let-7a and let-7b on p38MAPK and PKB phosphorylation, IκB expression, and NF-κB nuclear translocation. A: Western-blot assays for NF-κB translocation; B: Percentage of NF-κB (p65) in nucleus; C: IκB protein levels; D: Western-blot assays for p38MAPK, pp38MAPK, pPKB, PKB and IκB protein expression; E: Relative ratio of pp38MAPK/p38MAPK; F: Relative ratio of pPKB/PKB. The values (mean ± SD from three independent experiments) are relative to negative control, which was set as 1. ^##^ *P*<0.01 vs. NC, *P<0.05; **P<0.01 vs. oxLDL.

As shown in Figure 6, oxLDL treatment increased the pp38MAPK/p38MAPK significantly (3.87 folds compared to NC, Fig. 6D, E), while it inhibited the phosphorylation of PKB (42.9% compared with NC, Fig. 6D, F). Let-7a and let-7b over-expression inhibited the pp38MAPK/p38MAPK ratio and increased the pPKB/PKB. Interestingly, we found let-7a over-expression promote the pPKB/PKB ratio dramatically. The pPKB/PKB ratio is slightly higher than the NC group. Since the phosphorylation of PKB related tightly with NO production, we hypothesize that the anti-apoptotic and NO-promote effects of let-7 were achieved by regulation of p38MAPK and PKB phosphorylation.

### Effects of let-7a and let-7b on IκBα expression, and NF-κB nuclear translocation

Fig. 6C, D showed a remarkable decrease in IκB in oxLDL treated endothelial cells, indicating that oxLDL may enhance the degradation of IκB. Let-7a and let-7b over-expression inhibits the degradation of IκB. We also found a significant increase in the nuclear distribution of NF-κB in oxLDL treated endothelial cells compared with the negative control group. The percentage of NF-κB in nuclear is increased to 1.78 folds after oxLDL treatment. Let-7a and let-7b over-expression significantly suppresses oxLDL-induced nuclear translocation of NF-κB (Fig. 6A, B).

## Discussion

The present study demonstrated that 1) LOX-1 is a target of let-7a and let-7b, and they inhibit the expression of LOX-1 by targeting the position of 310–316 in LOX-1 3′-UTR. Over-expression of let-7a and let-7b inhibit the expression of LOX-1, while inhibition of let-7a/7b increase the LOX-1 expression; 2) In oxLDL treated endothelial cells, the let-7a and let-7b expression is suppressed in a time-dependent and concentration-dependent manner; 3) let-7a and let-7b over-expression inhibited oxLDL-induced endothelial cell apoptosis and dysfunction; 4) The effects of let-7a and let-7b on endothelial cells are partly obtained through LOX-1/ROS/p38MAPK/NF-κB signal pathway and LOX-1/ROS/PKB/eNOS pathway.

LOX-1 was first identified by Sawamura in 1997 [5]. It is expressed in endothelial cells, macrophages, vascular smooth muscle cells and platelets [6], [20]. LOX-1 can be up-regulated by many factors such as oxLDL, AngII, shear stress, and advanced glycation end products (AGEs) [21]. The up-regulation or activation of LOX-1 is thought to play important roles in pathogenesis of cardiovascular diseases, such as atherosclerosis, coronary artery diseases, and myocardial infarction [4]. Factors which suppress LOX-1 are thought to be beneficial to the cardiovascular systems. For example, anti-LOX-1 antibody treatment rescues the endothelium-dependent vasodilation in atherosclerotic ApoE knockout mice [10]. LOX-1 knockout in mice leads to a reduction in atherogenesis, proinflammatory and prooxidant signals [11]. SIRT1, an NAD(+)-dependent class III histone deacetylase, was also discovered to suppress LOX-1 expression, thus playing a protective role in atherosclerosis [12]. However, the precise regulatory mechanisms and other regulatory factors of LOX-1 are still unclear. The negative regulatory roles of microRNAs in gene expressions are well documented in recent years. Previous studies have demonstrated the inhibitory effects of let-7g on LOX-1, while our pilot studies also found that LOX-1 was a target for let-7a, let-7b, let-7c and miR-98 (data not shown). Since let-7a and let-7b are more abundantly expressed in HUVECs, we have selected these two let-7's for further studies. In the present study, the luciferase assays showed the inhibition effects of let-7a and let-7b on LOX-1 3′-UTR reporter plasmids by directly targeting 310–316 region in LOX-1 3′-UTR. In addition, the endothelial cell endogenous levels of LOX-1 mRNA and protein were inhibited by the over-expression of let-7a and let-7b. These results validate LOX-1 as one of the targets of let-7a and let-7b in endothelial cells. This suppressive effect of let-7a and let-7b on LOX-1 might be beneficial for cardiovascular systems.

Let-7 family, following lin-4, is the second miRNAs found. The let-7 family has 9 members, namely, let-7a, let-7b, let-7c, let-7d, let-7e, let-7f, let-7g, let-7i and miR-98. Previous studies have identified let-7 as a tumor suppressor, which is down-regulated or lost in many human cancers [22]. More recently, the roles of let-7 in cardiovascular biology and disease have received significant attentions. Recently, the functions of let-7 in the cardiovascular system have drawn more and more attentions [14]. They could serve as biomarkers, therapeutic targets or be used as drugs directly. Previous studies have reported the functions of let-7g in vascular smooth muscle cells [17]. However, the effects of other let-7 members on cardiovascular systems are still unclear. Thus our studies investigate the effects of let-7a and let-7b on oxLDL treated endothelial cells. In present part of the study, we found a time- and concentration-dependent decrease of let-7a and let-7b after oxLDL treatment, indicating that these two let-7's may participate in oxLDL-induced endothelial cell injuries. Since we have found that LOX-1 is a target of let-7a and let-7b, we therefore speculate the oxLDL-induced up-regulation of LOX-1 might be partly due to the down-regulation of let-7a and let-7b.

It is well known that the oxLDL injury causes endothelial cell apoptosis, which can in turn lead to a reduction of vascular integrity, deposition of lipids, invasion of vascular smooth muscle cells, migration of monocytes, and formation of atherosclerotic plaque [23]. Therefore, inhibition of endothelial cell apoptosis will prevent atherogenesis. In the present study, we found oxLDL treatment increased the apoptotic rate to 7.92 fold compared with control. Let-7a and let-7b over-expression inhibited the apoptotic rate, implying that let-7a and let-7b exhibit anti-apoptotic effects. And in oxLDL-damaged endothelial cells, the increase of apoptosis may be partly due to the down-regulation of let-7a and let-7b.

Vascular endothelium releases many vasoactive molecules to regulate the vascular tone, coagulation, fibrinolysis and leukocyte adherence [24], [25]. Among these molecules, NO is considered to be a key factor in vascular protective actions including mediation of vasodilatation, inhibition of thrombosis and suppression of inflammatory response [24]. The impaired endothelium-dependent relaxation caused by the loss of NO bioactivity is believed to be a key event in endothelial dysfunction [26]. NO is produced by eNOS, which is expressed in endothelial cells. Researches showed that in oxLDL-induced endothelial cells injuries, the expression of eNOS and the generation of NO were decreased [27]. Thus, protecting endothelial cells to express eNOS and produce NO will be beneficial to endothelium. The present study investigated the effects of let-7a and let-7b on NO and eNOS expression, and found let-7a and let-7b inhibited the oxLDL-induced NO and eNOS decrease. Moreover, let-7a or let-7b over-expression alone promoted the NO production. These results indicate that let-7a and let-7b achieved their protective effects through both oxLDL dependent and independent ways. The eNOS and NO retaining function may be the basis for the protective effects of let-7a and let-7b. The decreases in eNOS expression result in the deficiency of NO, which then feeds back and potently induces LOX-1 expression, leading to the augmented uptake of modified LDL by endothelial cells, and thus contributes to endothelial lipidosis and initiates early atherosclerosis [28]. The NO promoting effects of let-7 may attenuate the feedback, and contribute to their protective effects.

In the exploration of the mechanisms of the effects of let-7a and let-7b on cell apoptosis and NO production, we found two signaling pathways playing key roles in oxLDL-induced endothelial cell injuries: the LOX-1/ROS/p38MAPK/NF-κB signal cascade and the LOX-1/ROS/PKB/eNOS signal cascade [29], [30], [31]. In endothelial cells, oxLDL activates LOX-1, promotes ROS production and subsequently induces p38MAPK phosphorylation and PKB dephosphorylation, and the p38MAPK phosphorylation then induces NF-κB activation. P38MAPK and NF-κB activation results in the up-regulation of pro-inflammatory factors. And PKB dephosphorylation results in down-regulation of eNOS in response to oxLDL [29], [30], [31]. The present study found a significant inhibition of let-7a and let-7b on ROS production. ROS was thought to be the upstream of p38MAPK and PKB [9]. We also found an inhibition of let-7a and let-7b on oxLDL-induced p38MAPK phosphorylation and PKB dephosphorylation. Since PKB is thought to be the upstream of eNOS, our results may imply the induction of let-7a and let-7b on NO may achieve through PKB. Moreover, the overproduction of ROS directly inactivates the NO, the inhibitory effects of let-7a and let-7b on oxLDL-induced ROS overproduction may also contribute to the NO increase in the present study. NF-κB has been reported to be a multifunctional transcriptional factor, which regulates the expression of many genes [32]. It was also reported to be the downstream of p38MAPK [9]. The phosphorylation of p38MAPK induced the activation of NF-κB, which including the dissociation of NF-κB and IκBα in cytoplasm, and the subsequent nuclear translocation of NF-κB subunit p65 and p50 [33]. In nucleus, NF-κB presents as a primary p65/p50 heterodimer and binds directly to its cognate DNA sequence and interferes with the transcription of target genes [33]. Many studies have demonstrated the relationship of cell apoptosis and NF-κB activation [34], [35], [36]. Inhibition of NF-κB activation may be an effective method to anti-apoptosis. In the present part of study, we found inhibitory effects of let-7a and let-7b on oxLDL-induced IκB decrease and NF-κB activation. These results support the hypothesis that let-7a and let-7b might inhibit p38MAPK/NF-κB signal transduction to prevent endothelial apoptosis. Moreover, NF-κB activation also up-regulates LOX-1 expression and form a positive feedback loop [37]. Let-7a and let-7b also attenuate this positive feedback loop by preventing the activation of NF-κB.

In conclusion, let-7a and let-7b inhibited oxLDL-induced endothelial cell apoptosis and dysfunction by directly targeting LOX-1 and by inhibition of ROS overproduction, eNOS downregulation and subsequently NO production. p38MAPK phosphorylation, PKB dephosphorylation, NF-κB translocation and IκB degradation are associated with the effects of let-7a and let-7b. The present study may provide a new insight into the protective properties of let-7a and let-7b in preventing the endothelial dysfunction associated with cardiovascular disease, such as atherosclerosis.
